# Intestinal anisakiasis with severe intestinal ischemia caused by extraluminal live larvae: a case report

**DOI:** 10.1186/s40792-020-01033-2

**Published:** 2020-10-01

**Authors:** Kengo Shibata, Yuichi Yoshida, Yoichi Miyaoka, Shin Emoto, Tomoaki Kawai, Seiji Kobayashi, Kazuhiro Ogasawara, Akinobu Taketomi

**Affiliations:** 1 Division of Surgery, Japan Organization of Occupational Health and Safety, Kushiro Rosai Hospital, 13-23 Nakazono-cho, Kushiro City, Hokkaido 085-8533 Japan; 2 Department of Gastroenterological Surgery I, Hokkaido University Graduate School of Medicine, Kita 15, Nishi 7, Kita-ku, Sapporo, Hokkaido 060-8638 Japan

**Keywords:** Live larva, Extraluminal, Intestinal resection, Anisakiasis, Laparoscopic exploration

## Abstract

**Background:**

Anisakiasis is a parasitic infection caused by Anisakis worms found in raw fish. Most cases of anisakiasis occur in the stomach and rarely occur in the intestine. It is extremely rare for live larvae to break through the intestine into the mesentery and cause severe intestinal ischemia. Anisakiasis can be treated conservatively, because the larvae will die in approximately 1 week, but, sometimes, a serious condition can arise, as in this case. We report the first case of extraluminal anisakiasis in which a live Anisakis worm caused severe intestinal ischemia.

**Case presentation:**

The patient was a 26-year-old woman who ate squid a week prior. She had abdominal pain and was admitted to our emergency department. On physical examination, abdominal guarding and rebound tenderness were present in her lower abdomen. Contrast-enhanced computed tomography showed ascites, the whirl sign, localized submucosal edema of the intestinal wall, and a dilated small bowel segment with edema. We suspected the strangulated small bowel obstruction based on the CT-scan findings. To rule out the strangulated small bowel obstruction, laparoscopic exploration was performed. Bloody ascites in the pouch of Douglas and severe inflammation in 20 cm of the ileum were observed. An Anisakis larva had perforated the intestinal wall and was found alive in the mesentery. The ileum had developed a high degree of ischemia, so the affected section was resected. Histopathological examination revealed that the Anisakis worm body was in the inflamed mesentery and caused a high degree of ischemia in the intestinal tract. The patient was discharged 9 days after surgery.

**Conclusions:**

A living Anisakis larva punctured the mesentery of the small intestine, resulting in severe intestinal ischemia. As seen in this case, intestinal anisakiasis may cause serious symptoms, and a low threshold for performing diagnostic laparoscopy for the early diagnosis of bowel ischemia secondary to anisakiasis can be useful in determining the definite diagnosis and indications for resection.

## Background

Gastrointestinal anisakiasis has become common worldwide with the spread of raw fish consumption [1–3]. Approximately 20,000 cases of anisakiasis are found around the world annually, of which 90% involve the stomach, and only 4–5% involve the small intestine [4, 5]. The low incidence of anisakiasis in the small intestine is because Anisakis are easily found in the stomach endoscopically, thus preventing Anisakis migration into the small intestine [6]. Moreover, it is extremely rare that the Anisakis penetrates the digestive tract, and in only one case, a worm that was alive was found accidentally found during bilateral ovarian cystectomies [7]. It is noteworthy in this case that the Anisakis was found not only at extraluminally but also alive in the mesentery 1 week after the ingestion. We report the first case of severe ischemia in the ileum caused by a live worm body puncturing the mesentery and forming granulation.

## Case presentation

**Fig. 1:**
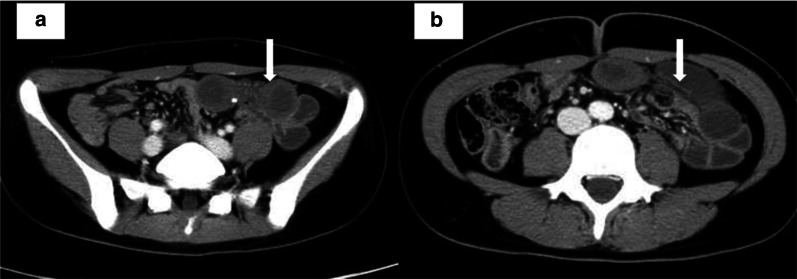
Contrast-enhanced CT findings. Contrast-enhanced CT revealed submucosal edema of the intestinal wall (arrow) and a sustained contrast effect (**a**). A whirl sign (arrow) was present, and strangulated small bowel obstruction was suspected (**b**)

The patient was a 26-year-old woman with a medical history of only asthma in childhood. She ate a sushi roll with squid a week ago and then had abdominal pain and was admitted to our emergency department. On physical examination, no allergic reaction, such as hives or bronchospasm, was observed, and her lower abdomen was flat and hard with tenderness and guarding. Laboratory examinations showed an elevated white blood cell count of 17,160/μl (leukocyte fraction could not be examined after hours in our hospital). Laboratory examinations showed increases in IgE-RIST levels (0.89 UA/ml; normal range < 0.34 UA/ml). There were no other abnormal findings. Contrast-enhanced computed tomography (CT) showed ascites, the whirl sign, localized submucosal edema of the intestinal wall, and a dilated small bowel segment with edema (Fig. 1).

We suspected the strangulated small bowel obstruction based on the CT-scan findings.

**Fig. 2:**
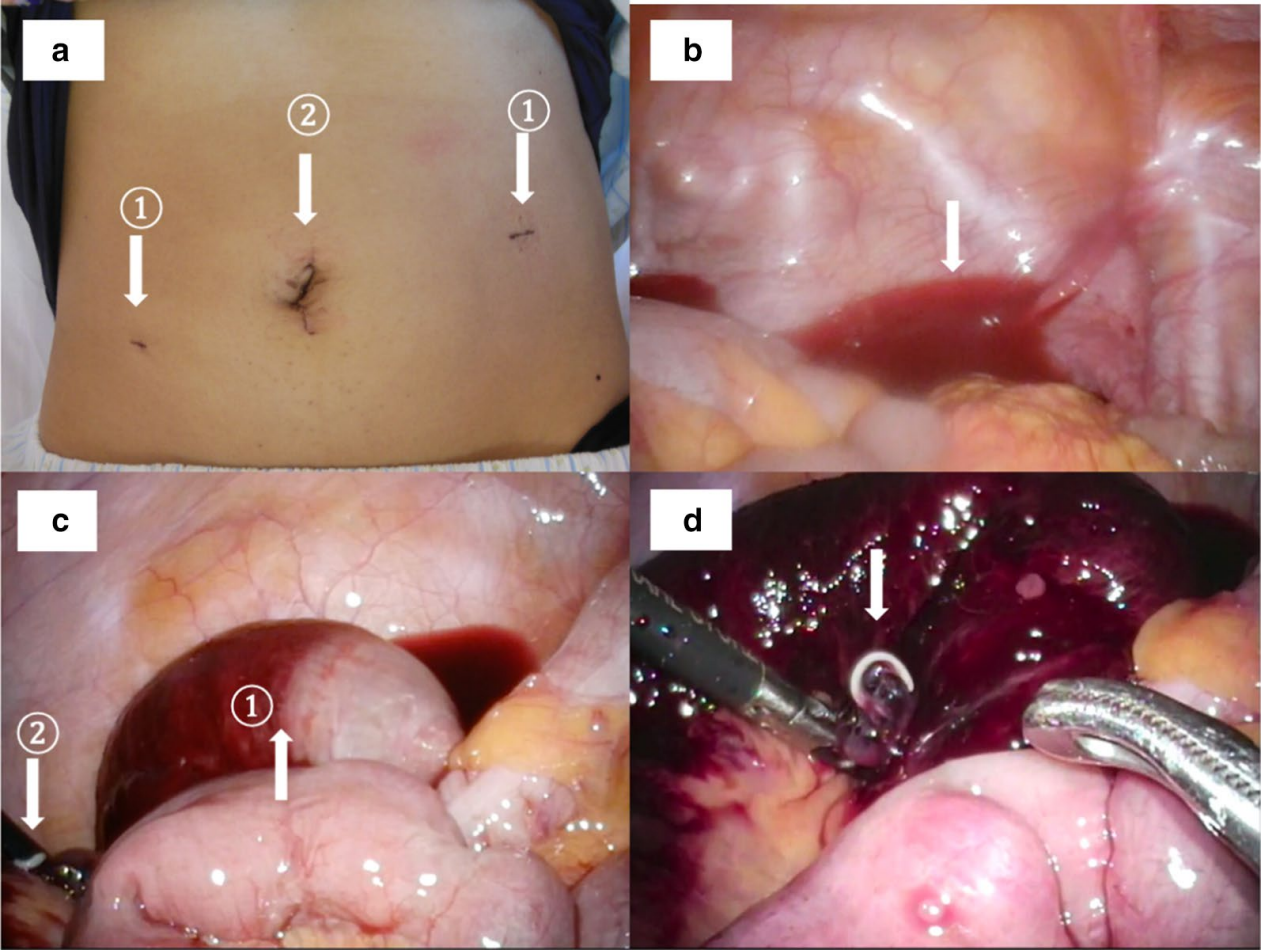
Findings during operation. **a** Trocar arrangement in this procedure. Two 5 mm ports were placed on both sides ①. The 12 mm port was placed in the navel, and a small incision was made to resect the small intestine ②. **b**–**d** Laparoscopic exploration findings. **b** There were bloody ascites in the pouch of Douglas. **c** Severe local circulatory abnormalities were found in the ileum. There were no findings that caused strangulation ①. The Anisakis worm was found on the edge of screen ②. **d** A live larva had pierced the mesentery of the ileum

The presence of acute abdominal guarding and the CT-scan findings indicated that strangulated small bowel obstruction could not be ruled out completely, and a laparoscopic examination was performed. A 12 mm port was placed in the navel, and two 5 mm ports were placed in the left and right flanks. Bloody ascites in the pouch of Douglas and severe inflammation and bloody ascites were observed in 20 cm of the ileum. Neither the oral side nor the anus side of the inflamed intestinal tract was narrowed, and the intestinal tract suddenly changed from a normal appearance to an inflammatory appearance. No cords that caused strangulation were observed. The Anisakis worm body was found to have punctured through to the outside of the intestine, and the worm was found alive and moving in the mesentery. The umbilical wound was extended to 2 cm, and the ileum was placed outside the body cavity. Strong inflammation of the intestinal tract was observed. A segmental resection of 20 cm of the inflamed bowel, located 40 cm from the ileum end, was performed. The small intestine was reconstructed with a functional end-to-end anastomosis (Fig. 2).

**Fig. 3:**
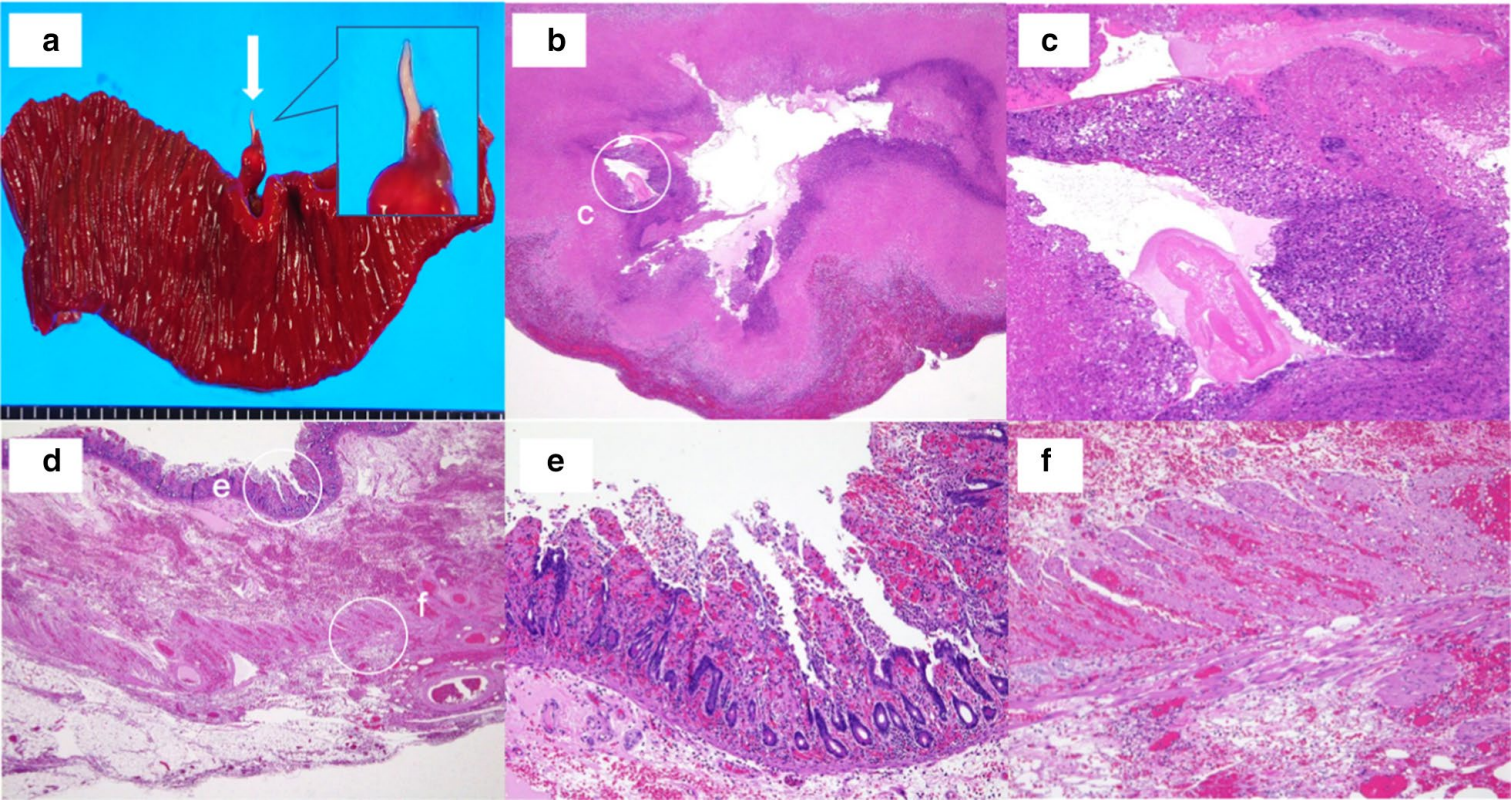
Excised specimen and pathological findings. **a** Gross appearance of the excised specimen. The small intestinal mucosa was edematous and thickened, and a live Anisakis larva (arrow) was seen burrowing into the intestinal wall. A live Anisakis larva (arrow) is observed in the mesentery, along with severe circulatory disturbance and edematous of the intestinal wall. There was severe damage, but no obvious perforation was found in the intestinal wall. **b**, **c** Histopathology of the larva in the mesentery with hematoxylin and eosin (H&E) staining, **b** 2×, and (c) 10× magnification. The Anisakis larva was visually recognized with cuticular fragments. Eosinophil infiltration was not prevalent, but neutrophils were often phagocytosed, and granulation was observed. **e**–**f** Histopathology of the small intestine with H&E staining, and (**d**) 2× and (**e**, **f**) 10× magnification. The mucous membrane was extensively degraded and showed a ghost-like appearance. The muscle layer showed bleeding and severe circulation abnormalities

The resected specimen was associated with a high degree of ischemia along the entire thickness, and the living Anisakis worm body was confirmed in the mesentery. Histological examination revealed the presence of parasites in the mesentery, phagocytosis of neutrophils in the surrounding area, and granuloma formation. The mucous membrane of the intestinal tract was extensively degraded, and the muscular layer showed severe bleeding. Eosinophilic infiltration was not remarkable, and the phagocytosis of neutrophils was often observed (Fig. 3).

The patient was discharged 9 days after surgery.

## Discussion

In Japan, there is a culture of eating raw fish, which can lead to gastrointestinal anisakiasis. Yasunaga et al. reported that the annual incidence of bowel anisakiasis in Japan is estimated to be approximately 3.0 per 1 million people per year, and the associated ileus, bleeding, and intussusception rates are 50.7%, 8.0%, and 2%, respectively. Among all patients with anisakiasis, 7% undergo surgical treatment [8]. Anisakiasis in the small bowel is associated with many symptoms, such as obstruction and abdominal pain, so it is difficult to make a definitive diagnosis and distinguish this condition from acute abdomen [9–11]. It has been reported that leukocytosis, eosinophilia, and the anti-Anisakis antibody titer are useful for diagnosing small intestinal anisakiasis [6, 12, 13]; however, these factors may not always be useful for diagnosing acute abdomen, because they cannot be tested in an emergency department, it takes time to produce the results, and false-negative and false-positive results are possible. In this case, the IgE-RIST level changed from 0.89 UA/ml on the operation day to 1.44/1.23/0.81 UA/ml (on days 7/15/36 after the operation). The elevation of the IgE-RIST level sometimes helps to confirm the postoperative diagnosis; however, it takes a few days until the result is returned. Among the other preoperative diagnosis tools, various imaging techniques for diagnosing anisakiasis have been reported. Shibata et al. reported that most intestinal anisakiasis causes marked submucosal edema of the small bowel without showing complete intraluminal occlusion, ascites, or increased attenuation of the adjacent fat on CT [14]. Although CT and ultrasound have been reported to be useful in the diagnosis of intestinal Anisakis, it is often very difficult to distinguish anisakiasis from strangulated small bowel obstruction [10, 14–16]. Therefore, surgical exploration should be considered for diagnostic purposes.

Studies have reported that the time from the consumption of raw fish to the development of abdominal pain is 12–24 h for gastric anisakiasis and 5–7 days for intestinal anisakiasis [6, 17–19]. This case was caused by eating squid in a sushi roll 1 week prior. To diagnose intestinal Anisakis, it may be necessary and useful to obtain a diet history that spans more than 1 week [8, 17]. There is no doubt that patient interviews are very important. However, it may be difficult to obtain such details in the examination of acute abdomen. This eating history was acquired after the patient’s condition improved 2 days after the operation.

In the patients with anisakiasis, the parasites can be retained in the intestinal lumen and remain alive for up to 1 week at most, so conservative treatment is possible [4]. However, the small intestine has a weaker wall than the stomach, and perforation can occur in patients with intestinal anisakiasis. Matsuo et al. reported that two patients needed surgical treatment on the 23rd and 35th days in the hospital [20]. Allison et al. also reported that a patient needed surgical treatment 14 days after being discharged home [21]. Therefore, it should always be considered that the need for therapeutic intervention may be delayed.

In this case, the patient suffered from severe intestinal ischemia, and timely observation of the abdomen with surgery was important, as well as clinical findings such as abdominal guarding. Moreover, laparoscopic exploration is minimally invasive and imposes a small burden on the patient. This approach is also very useful as it requires minimal additional incisions when intestinal resection is required [22]. However, if the operation is delayed, severe small bowel obstruction will occur and laparoscopic surgery may not be possible, so careful observation is necessary.

This is a very rare case in which a living Anisakis perforated the mesentery, causing severe intestinal ischemia with bloody ascites. Based on the pathological findings, the intestinal tract may have become highly ischemic due to the direct inflammation caused by the Anisakis perforating the intestinal wall into the mesentery, the collapse of the mesenteric blood vessels, vasoconstriction due to allergic reaction, and ischemia from the granuloma formation process. The pathological results indicated that intestinal necrosis was starting and that resection was appropriate. In this case, the clinical findings of strong abdominal symptoms and severe local circulatory injury and laparoscopic findings of bloody ascites were the indications for resection to treat this patient with intestinal anisakiasis.

## Conclusion

We experienced a case of small bowel anisakiasis in which a living worm perforated the intestine, inflamed the mesentery, and caused ischemic damage. Intestinal anisakiasis can be considered a serious condition, and close observation by surgeons may be necessary. Laparoscopic exploration is useful in diagnosing intestinal anisakiasis and guiding the decision for bowel resection.

## Data Availability

The data are not available for public access because of patient privacy concerns, but are available from the corresponding author on reasonable request.

## Abbreviation

CT: Computed tomography
